# Age-dependent frequency of unconventional T cells in a healthy adult Caucasian population: a combinational study of invariant natural killer T cells, γδ T cells, and mucosa-associated invariant T cells

**DOI:** 10.1007/s11357-022-00515-5

**Published:** 2022-01-17

**Authors:** Parvind Singh, Marianna Szaraz-Szeles, Zoltan Mezei, Sandor Barath, Zsuzsanna Hevessy

**Affiliations:** Department of Laboratory Medicine, Faculty of Medicine, University of Debrecen, Nagyerdei krt. 98, 4032 Debrecen, Hungary

**Keywords:** Unconventional T cells, iNKT cells, γδ T cells, MAIT cells, Frequency, Reference range, Age dependent

## Abstract

Unconventional T cells show distinct and unique features during antigen recognition as well as other immune responses. Their decrease in frequency is associated with various autoimmune disorders, allergy, inflammation, and cancer. The landscape frequency of the unconventional T cells altogether (iNKT, γδ T, and MAIT) is largely unestablished leading to various challenges affecting diagnosis and research in this field. In this study, we have established the age group–wise frequency of iNKT, γδ T, and MAIT cells altogether on a total of 203 healthy adult samples of the Caucasian population. The results revealed that iNKT cells were 0.095%, γδ T cells were 2.175%, and MAIT cells were 2.99% of the total T cell population. γδ and MAIT cell frequency is higher in younger age groups than elderly; however, there is no statistically significant difference in the frequency of iNKT cells. Furthermore, γδ and MAIT cells were negatively correlating with age, supporting immunosenescence, unlike iNKT cells. Our finding could be used for further age-wise investigation of various pathological conditions such as cancer and their prognosis, autoimmune diseases and their pathogenicity.

**Supplementary Information:**

The online version contains supplementary material available at 10.1007/s11357-022-00515-5.

## Introduction

Unconventional T cells do not fit in the paradigm with conventional T cells, which are major histocompatibility complex (MHC) dependent and recognize peptide antigens. Instead, unconventional T cells are non-MHC restricted T cells recognizing non-peptide, non-polymorphic antigen-presenting molecules and containing αβ and γδ T cell receptors. Usually, these cells represent a minor T cell population in peripheral blood (PB) of healthy individuals [1]. Deficiency and depletion of these cells are associated with autoimmune disorders, inflammations, and cancers [2].

The T cell receptor (TCR) invariant natural killer T cells (iNKT) recognize lipid antigens presented by CD1d [3], which is a non-classical MHC class I–like antigen–presenting molecule. Several lipid antigens have been reported from bacterial pathogens [4], and various types of hematological malignant cells [5, 6] and solid tumor cells [7, 8]. Cancer immunotherapy became a potential target to treat cancer in the last decade and iNKT cells showed unique features and multiple mechanisms to target cancer cells, i.e., direct killing of CD1d^+^ tumors, and adjuvant effects by recruiting NK cells and dendritic cells, further activating cytotoxic T lymphocytes and inhibition of tumor-associated macrophages [9]. Several clinical trials prove the effectiveness of iNKT cells to target hematological malignancies and tumor cells [9–11]. The lower frequency of iNKT cells is associated with various kinds of pathological conditions including type-1 diabetes, rheumatoid arthritis, systemic lupus erythematosus, multiple sclerosis, Parkinson’s disease, Lyme disease, tuberculosis, solid tumors, and various types of hematological malignancies [12–14].

TCR γδ cells are abundant in the skin and gut tissues which suggests their role in the first line of defense against infections. γδ T cells recognize the Mycobacterium tuberculosis and tetanus toxoid as a primary defense against these infections [15, 16] and are associated with various other pathological conditions such as infections including bacterial, protozoal, and viral, autoimmune disorders, transformed or cancer cells [17, 18]. γδ T cells are also involved in immune surveillance against cancer development including hematological diseases and solid tumors [19]. γδ T cells do not require conventional antigen processing and presentation through MHC molecules. Instead, they recognize the cells that have been exposed to low molecular weight, phosphate-containing non-peptide antigens (phospho-antigens), which are produced by bacterial specific isoprenoid biosynthesis pathways and some synthetic phosphate analogues are also found called synthetic phospho-antigens [20–22]. Microbial, as well as mammalian, cells share non-peptide ligands; these ligands are phosphorylated metabolites of thymidine-containing nucleotide conjugate that appears during the salvage pathway and are overexpressed by damaged or stressed cells [21]. MHC class I–related molecule MIC-A and MIC-B are stress-induced antigens and are expressed on various epithelial tumors [23]. These stress-induced MIC-A or MIC-B expressing cells can be recognized via natural killer group 2 member D (NKG2D) activating receptors of γδ T cells [24]. Various types of melanoma, lymphoma, leukemia, and carcinoma express a large number of phosphorylated mevalonate metabolites [25] and it was shown that γδ T cells are capable of killing these cells by recognizing phospho-antigens [26]. In addition, γδ T cells are being explored in cell-based immunotherapy to target cancer cells [27–29].

Mucosa-associated invariant T cells (MAIT) recognize microbial riboflavin-derivative antigens presented by non-polymorphic MHC class I–like protein MR1 [30]. TCR repertoires of MAIT cells are conserved across the species and they are activated by both TCR-dependent and independent mechanisms and produce innate-effector response upon activation [31]. These cells are in abundance in the liver representing up to 45% of liver T cells [32] and up to 10% of T cells in peripheral blood [33]. Microbial infections, which can utilize riboflavin biosynthesis pathways with 5-A-RU (5-amino-6-D-ribitylaminouracil), activate MAIT cells [31] and the pyrimidine derivatives 5-OP-RU (5-(2-oxopropylideneamino)-6-D-ribitylaminouracil) loaded into MR-1 tetramer are being used for identification of MAIT cells in vitro as well as for activation of MAIT cells in vivo [34, 35]. MAIT cells play important role in bacterial and viral infections and an association was shown with riboflavin synthesizing bacteria and a few non-riboflavin synthesizing bacterial, viral, and fungal infections [36–39]. Along with it, they have an association with autoimmune and immune-mediated disorders such as multiple sclerosis, coeliac disease and inflammatory bowel diseases, systemic lupus erythematosus (SLE) and arthritis, Sjögren’s syndrome, asthma and chronic obstructive pulmonary disease, psoriasis, type 1 diabetes, type 2 diabetes and obesity, and liver diseases [40, 41]. A decrease in the frequency of circulating MAIT cells was associated with various solid tumors such as colorectal cancer, hepatocellular carcinoma, lung cancer [42–45], and hematological malignancy such as multiple myeloma [46].

Unconventional T cells (iNKT, γδ T, and MAIT) have been studied in a wide variety of pathological disorders including infections (viral and bacterial), immune-mediated diseases, autoimmune disease, and malignancies (solid and hematological) and much more research continues to explore the role of unconventional T cells; however, the reference range of these cells is not properly established on a larger group of samples with the categorization of age group–based discrepancies. In this cohort, we have established the reference range using 203 human adult peripheral blood samples to measure the frequency of iNKT, γδ T, and MAIT cells, along with age group–wise comparison of these cells.

## Material and method

### Exclusion criteria

Upon receipt the samples were evaluated according to the exclusion criteria defined by SENIEUR protocol [47], briefly, any infections (< 6 weeks), inflammation (acute and chronic), autoimmune disorder, human immunodeficiency virus (HIV) infection, hepatitis B virus (HBV) infection, hepatitis C virus (HCV) infection, SARS CoV-2 infection (< 1 year), diabetes mellitus, immunosuppressive drugs, alcoholism and drug abuse, current pregnancy or breastfeeding, malignancies (any form), immunomodulatory therapy, vaccination (< 6 weeks), and other conditions which influence immune system were excluded from the study.

### Study population

A prospective study was designed and conducted between November 2020 and May 2021 to define the reference range in healthy adults of unconventional T cells subsets (iNKT cells, γδ T cells, and MAIT cells) in the Caucasian population residing in central Europe from Hungary.

Four different age groups were defined and samples were collected separately for each group (Table 1). A total of 203 samples were included (94 males and 109 females) and 64 samples were excluded based on exclusion criteria. According to the 2008 guideline of the Clinical Laboratory Standards Institute, we determined the frequency of unconventional T cells in different age groups (18–35; 36–50; 51–65; 66–90 years) in order to establish age-dependent reference ranges [48].

**Table 1:** Number of healthy adult population included in the study

	Age groups (years)			
	18–35	36–50	51–65	66–90
Total samples included (*n*)	56	77	52	18
Mean age ± standard deviation	28 ± 5	43 ± 4	57 ± 4	72 ± 4

### Blood sample collection and cell counting

Peripheral venous blood (3 mL) samples were obtained from the hematology division; these samples were collected in a BD vacutainer tube (Becton Dickinson, San Jose, CA, USA) containing tri-potassium ethylene-diamine tetra acetic acid (K3-EDTA) through venipuncture. A complete blood count (CBC) was performed using a six-part hematology analyzer Siemens ADVIA® 2120i (Siemens Healthcare GmbH, Erlangen, Germany).

### Sample stability

To establish sample stability for these unconventional T cells, a total of ten samples were analyzed for 96 h, where measurement was repeated after every 24 h.

### Flow cytometry

Multiparametric eight color flow cytometric experiment using the following pre-titrated mouse anti-human monoclonal antibodies from Beckman Coulter (Brea, CA, USA), Dako (Glostrup, Denmark), Biolegend (San Diego, CA, USA), BD/Pharmingen (Franklin Lakes, NJ, USA), and Exbio (Prague, Czech Republic): anti-human CD16 (clone 3G8; BC/IOT), anti-human TCR Vγ9 (clone B3; Biolegend), conjugated with fluorescein-isothiocyanate (FITC); anti-human CD56 (clone MOC-1; Dako), anti-human CD7 (clone 8H8.1; BC/IOT), conjugated with phycoerythrin (PE); anti-human TCR Vα24-Jα18 (clone 6B11; Biolegend), anti-human TCR Vδ2 (clone B6; Biolegend), anti-human TCR Va7.2 (clone 3C10; Biolegend), conjugated with peridinin chlorophyll protein/Cynine 5.5 (PerCP/Cy5.5); anti-human TCR Va24 (clone C15; BC/IOT), anti-human CD161 (clone 191B8; BC/IOT), conjugated with PE-Cynine 7 (PC7); anti-human CD3 (clone SK7; BD), conjugated with allophycocyanin (APC); anti-human CD8 (clone SK1; BD), conjugated with allophycocyanin cyanine 7 (APC-H7); anti-human CD4 (clone RPA-T4; BD), conjugated with pacific blue (PB); and anti-human CD45 (clone HI30; Exbio), conjugated with pacific orange (PO).

These monoclonal antibodies were combined in three different tubes to quantify and characterize iNKT cells, γδ T cells, and MAIT cells. Stain, lyse, and wash protocol was used to perform cell surface staining. Briefly, 100 µL of PB was mixed with an appropriate cocktail of mAbs in a FACS tube and incubated for 15 min in dark at room temperature (20–22 °C). Followed by 1 mL of 1 × RBC lysis buffer (BD FACS™ lysing solution) was added and incubated for 10 more minutes. Upon incubation, tubes were washed once with 1 mL of phosphate-buffered saline and centrifugation at 1500 rpm for 5 min, and the pellet was finally resuspended in 400 µL of 1% paraformaldehyde. All samples were stained within 8 h of sample collection and upon flow cytometric staining, 1% paraformaldehyde (PFA) was added to fix the cells for acquisition and analysis. Sample acquisition was performed using BD FACSCanto II^TM^ (Franklin Lakes, NJ, USA) flow cytometer, and cytometer setup and tracking beads (CS&T) was measured on daily basis to keep performance tracking of equipment. External quality control assessment was also achieved by participating in the UK-NEQAS Leukemia immunophenotyping program. Tubes were acquired using a carousel setting with high acquisition speed and a stop gate was set on 300,000 events for each tube.

**Fig. 1:**
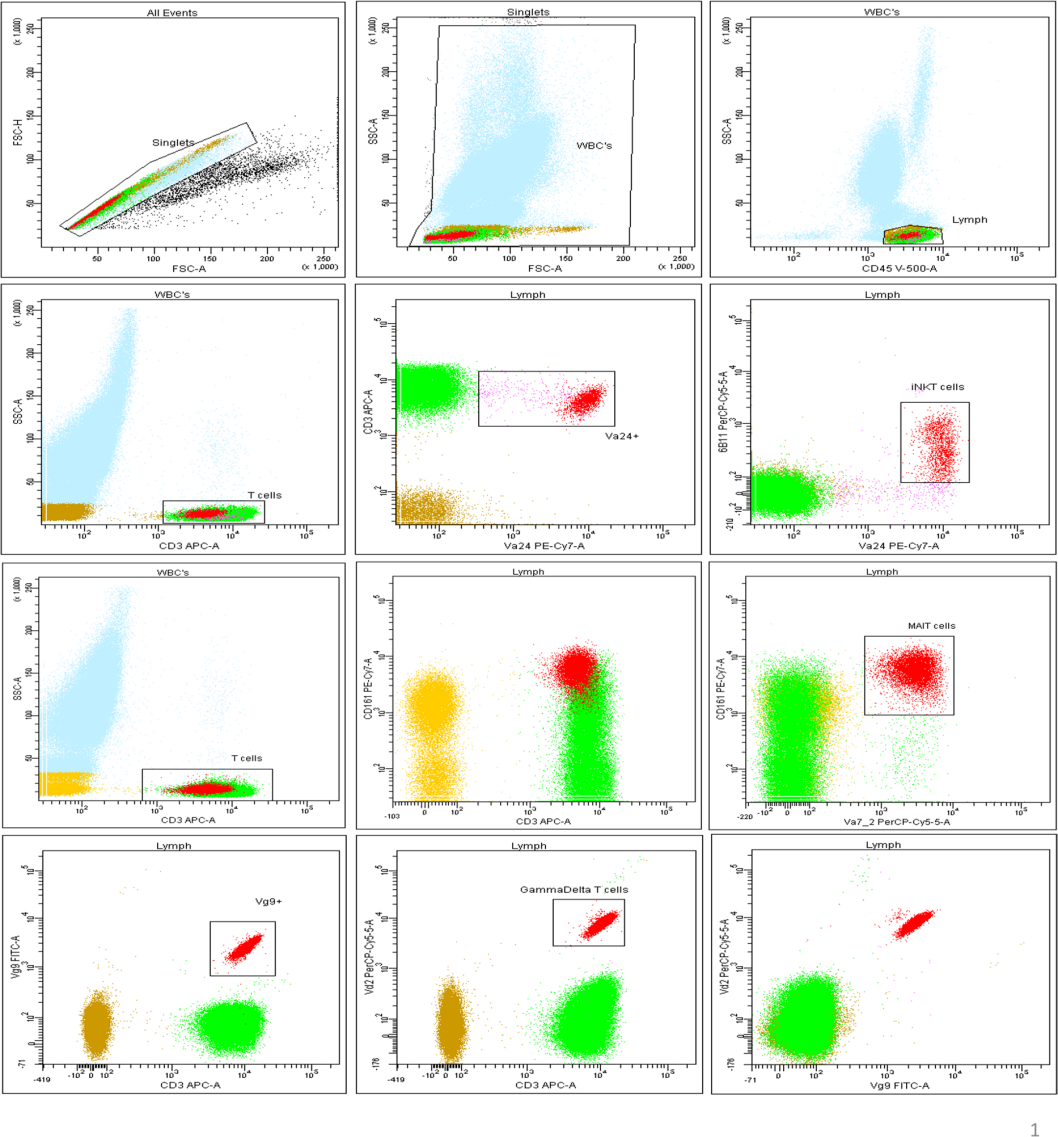
Dot plots and gating strategies obtained from multicolor flow cytometric panel of peripheral blood of a healthy adult donor, which includes iNKT cells, γδ T cells, and MAIT cells; universally, doublets were excluded via gating singlets on FSC-A vs FSC-H dot plot, followed by WBCs were gated on FSC vs SSC dot plot, and later lymphocytes were gated on CD45 vs SSC. iNKT cells gating strategy: T cells were gated on CD3 vs SSC dot plot, followed by Vα24 + iNK T cells were gated as Vα24 vs CD3 dot plot, later iNKT cells were gated as 6B11 vs Vα24 (among T cells among WBC). MAIT cells gating strategy: T cells were gated on CD3 vs SSC dot plot, followed by CD3 vs CD161 dot plot were taken to visualize CD161 bright signal, later MAIT cells were gated as Vα7.2 vs CD161 dot plot (among T cells and among WBC). γδ T cells gating strategy: Vγ9 cells were gated on CD3 vs Vγ9 dot plot (among T cells and among WBC), followed by γδ T were gated on Vδ2 vs CD3 (among Vγ9 and among T cells) also γδ T cells were visualized on Vδ2 vs Vγ9 dot plot

Monoclonal antibodies (Vα24Jα18), clone 6B11 specifically recognizes CDR3 loop of invariant human canonical Vα24Jα18 TCR α chain of iNKT cells [49] were defined as CD45 + /CD3 + / Vα24 + /Vα24-Jα18 (6B11) + , γδ T cells were defined as CD45 + /CD3 + /Vγ9 + /Vδ2 + , and MAIT cells were defined as CD45 + /CD3 + /Vα7.2 + /CD161bright + (Fig. 1). Flow cytometric data files (.fcs 3.0) were analyzed using FACSDiva version 6.1.3 software (BD Biosciences, San Jose, CA, USA). The following percentages were calculated: lymphocytes (among white blood cells, WBC); T cells (among lymphocytes and among WBC); iNKT cells and MAIT cells (among total T cells and among lymphocytes); and γδ T cells (among Vδ2, among Vγ9, and among total T cells). Absolute count of total iNKT cells, γδ T cells, and MAIT cells was calculated from the absolute WBC and lymphocyte counts (× 10^9^/L) obtained from CBC report of hematology analyzer using dual-platform method and values were expressed as cells/µL.

### Statistical analysis

All the values were entered directly into the spreadsheet and further exported to the GraphPad Prism statistical software v5.0 (GraphPad Software, San Diego, CA, USA) for statistical analysis. Normality of data distribution was assessed by Kolmogorov–Smirnov (KS) and Shapiro–Wilk normality tests. To compare the different age groups, a non-parametric Kruskal–Wallis test with Dunn’s post hoc test was used. In addition, linear regression analysis was performed between two continuous variables and the statistical significance of the findings was set at a *p*-value of less than 0.05.

## Results

### Population statistics

A total of 203 samples were included and 64 samples were excluded in the study according to exclusion criteria, age group–wise total number of sample distribution, mean age, and standard deviation are shown in Table 1.

### Sample stability

This experiment revealed that the aforementioned CD markers and unconventional T cells were stable for up to 4 days without any statistically significant change in the percentage of the populations (Fig s1).

### Descriptive statistics of unconventional T cells

PB unconventional T cells (iNKT, γδ, MAIT) along with absolute (WBC and lymphocyte) counts were analyzed, where median, minimum, maximum, 25–75% and 2.5–97.5% (reference range) of hematological counts and percentages, and their absolute number are also shown in Table 2. The median percentage of iNKT cells among T cells was 0.095% ranging from 0.007 to 4.94%, and the median absolute number of iNKT cells was 1.3 cells/µL, ranging from 0.1 to 63.5 cells/µL. The median percentage of γδ T cells among T cells was 2.175% ranging from 0.078 to 16.09% and the median absolute number was 30 cells/µL ranging from 1 to 249 cells/µL (Table 2). MAIT cell ratio among T cells was 2.99% ranging from 0.11 to 18.36% and the absolute number of MAIT cells was 42 cells/µL ranging from 2 to 261 cells/µL (Table 2). Reference range for percentages of unconventional T cells (iNKT, γδ T, and MAIT cells) among T cells was 0.01–1.52%, 0.31–11.94%, and 0.27–10.74% respectively.

**Table 2:** Descriptive statistics of unconventional T cells obtained from total healthy adult PB samples of Caucasian population; data analyzed as a whole

Method (equipment)	Parameter	Median (min–max)	25–75%	2.5–97.5%
Hematology analyzer	WBC (× 10^9^/L)	6.66 (3.76–12.35)	5.86–7.77	4.59–10.62
	Lymphocytes (× 10^9^/L)	2.04 (0.97–4.91)	1.71–2.42	1.08–3.39
Flow cytometer	T cell (% lymphocytes)	69.78 (45.92–84.42)	64.67–75.2	53.65–82.84
	iNKT cells (% T cell)	0.095 (0.007–4.94)	0.042–0.254	0.01–1.52
	γδ T cell (% T cell)	2.175 (0.078–16.09)	1.163–4.31	0.31–11.94
	MAIT cells (% T cell)	2.99 (0.11–18.36)	1.43–5.4	0.27–10.74
Dual platform	iNKT (cells/µL)	1.3 (0.1–63.5)	0.6–3.6	0.1–18.34
	γδ T cell (cells/µL)	30 (1–249)	17.75–63	4.07–158
	MAIT (cells/µL)	42 (2–261)	18–76	4–181

### Age group–based descriptive statistics

The median, minimum, maximum, the percentile 25–75% and 2.5–97.5% (reference range) of hematological counts (WBC and lymphocytes) and percentages of unconventional T cells (iNKT, γδ, MAIT) along with their absolute numbers in PB were analyzed according to their respective age groups and are shown in Table 3.

**Table 3:** Age group–wise descriptive statistics of unconventional T cells in healthy adult PB samples of Caucasian population

Age groups	Method (equipment)	Parameter	Median (min–max)	25–75%	2.5–97.5%
18–35	Hematology analyzer	WBC (× 10^9^/L)	6.79 (3.76–12.24)	5.88–7.83	3.91–11.49
		Lymphocytes (× 10^9^/L)	2.07 (1.02–4.91)	1.778–2.465	1.04–4.24
	Flow cytometer	T cell (% lymphocytes)	69.61 (49.93–83.53)	66.19–72.25	53.86–82.94
		iNKT cells (% T cell)	0.136 (0.007–4.94)	0.052–0.395	0.0074–4.47
		γδ T cell (% T cell)	3.785 (0.685–16.09)	1.97–5.938	0.80–14.56
		MAIT cells (% T cell)	5.025 (0.67–11.91)	3.038–7.218	0.793–11.39
	Dual platform	iNKT (cells/µL)	2.0 (0.1–63.5)	0.8–4.5	0.1–61.76
		γδ T cell (cells/µL)	56.5 (9–249)	26.25–86.25	9.85–238
		MAIT (cells/µL)	71 (10–183)	43.75–98.75	12.98–177
36–50	Hematology analyzer	WBC (× 10^9^/L)	6.66 (4.71–10.63)	5.91–7.62	4.79–10.13
		Lymphocytes (× 10^9^/L)	2.13 (1.05–3.05)	1.71–2.49	1.17–3.03
	Flow cytometer	T cell (% lymphocytes)	70.86 (53.59–83.01)	65.82–75.83	54.2–82.95
		iNKT cells (% T cell)	0.078 (0.01–2.60)	0.041–0.199	0.011–1.98
		γδ T cell (% T cell)	1.98 (0.11–11.97)	1.02–3.315	0.263–10.59
		MAIT cells (% T cell)	3.42 (0.3–18.36)	1.555–5.02	0.404–13.2
	Dual platform	iNKT (cells/µL)	1.1 (0.1–44.1)	0.55–2.5	0.195–19.88
		γδ T cell (cells/µL)	28 (1–203)	15–52.5	3.85–125
		MAIT (cells/µL)	45 (5–261)	23–77.5	5–224
51–65	Hematology analyzer	WBC (× 10^9^/L)	6.77 (4.59–12.35)	5.91–7.89	4.74–11.93
		Lymphocytes (× 10^9^/L)	2.04 (0.97–4)	1.715–2.313	0.976–3.93
	Flow cytometer	T cell (% lymphocytes)	67.79 (45.92–84.42)	61.4–76.17	48.22–83.71
		iNKT cells (% T cell)	0.108 (0.011–1.152)	0.039–0.208	0.012–0.994
		γδ T cell (% T cell)	1.66 (0.078–7.23)	0.95–2.86	0.153–6.98
		MAIT cells (% T cell)	1.85 (0.11–10.59)	0.642–2.378	0.13–10.29
	Dual platform	iNKT (cells/µL)	1.15 (0.1–13.2)	0.425–2.875	0.1–12.32
		γδ T cell (cells/µL)	23.5 (1–122)	13–48.25	1.65–119
		MAIT (cells/µL)	23.5 (2–102)	7.25–42.25	2.32–100
66–90	Hematology analyzer	WBC (× 10^9^/L)	6.31 (4.54–9.24)	4.90–7.37	4.54–9.24
		Lymphocytes (× 10^9^/L)	1.72 (1.2–3.56)	1.47–2.258	1.2–3.56
	Flow cytometer	T cell (% lymphocytes)	69.81 (52.45–81)	60.65–76.32	52.45–81
		iNKT cells (% T cell)	0.098 (0.011–0.816)	0.024–0.159	0.011–0.816
		γδ T cell (% T cell)	1.5 (0.11–15.32)	0.77–2.765	0.11–15.32
		MAIT cells (% T cell)	1.345 (0.2–13.2)	0.34–2.403	0.2–13.2
	Dual platform	iNKT (cells/µL)	1 (0.1–16)	0.275–2.15	0.1–16
		γδ T cell (cells/µL)	20 (1–160)	10–46	1–160
		MAIT (cells/µL)	16.5 (2–211)	4–28.75	2–211

In the age group 18–35, median percentage of iNKT cells among T cells was 0.136%, ranging from 0.007 to 4.94%, γδ T cells was 3.785% ranging from 0.685 to 16.09%, and MAIT cells was 5.025% ranging from 0.67 to 11.91%. The median absolute number of iNKT cells was 2 ranging from 0.1 to 63.5 (cells/µL), γδ T cells was 56.5 ranging from 9 to 249 (cells/µL), and MAIT cells was 71 ranging from 10 to 183 (cells/µL) respectively. Reference range for % of unconventional T cells (iNKT, γδ T, and MAIT cells) among T cells was 0.0074–4.47%, 0.80–14.56%, and 0.793–11.39% respectively (Table 3).

In the age group 36–50, median percentage of iNKT cells among T cells was 0.078%, ranging from 0.01 to 2.601%, γδ T cells was 1.98% ranging from 0.11 to 11.97%, and MAIT cells was 3.42% ranging from 0.3 to 18.36%. The median absolute number of iNKT cells was 1.1 ranging from 0.1 to 44.1 (cells/µL), γδ T cells was 28 ranging from 1 to 203 (cells/µL), and MAIT cells was 45 ranging from 5 to 261 (cells/µL) respectively. Reference range for % of unconventional T cells (iNKT, γδ T, and MAIT cells) among T cells was 0.011–1.98%, 0.263–10.59%, and 0.404–13.2% respectively (Table 3).

In the age group 51–65, median percentage of iNKT cells among T cells was 0.108%, ranging from 0.011 to 1.152%, γδ T cells was 1.66% ranging from 0.078 to 7.23%, and MAIT cells was 1.85% ranging from 0.11 to 10.59%. The median absolute number of iNKT cells was 1.15 ranging from 0.1 to 13.2 (cells/µL), γδ T cells was 23.5 ranging from 1 to 122 (cells/µL), and MAIT cells was 23.5 ranging from 2 to 102 (cells/µL). Reference range for % of unconventional T cells (iNKT, γδ T, and MAIT cells) among T cells was 0.012–0.994%, 0.153–6.98%, and 0.13–10.29% respectively (Table 3).

Finally, in age group 66–90, median percentage of iNKT cells among T cells was 0.098%, ranging from 0.011 to 0.816%, γδ T cells was 1.5% ranging from 0.11 to 15.32%, and MAIT cells was 1.345 ranging from 0.2 to 13.2%. The median absolute number of iNKT cells was 1 ranging from 0.1 to 16 (cells/µL), γδ T cells was 20 ranging from 1 to 160 (cells/µL), and MAIT cells was 16.5 ranging from 2 to 211 (cells/µL) respectively. Reference range for % of unconventional T cells (iNKT, γδ T, and MAIT cells) among T cells was 0.011–0.816%, 0.11–15.32%, and 0.2–13.2% respectively (Table 3).

### Age group–based analysis

**Fig. 2:**
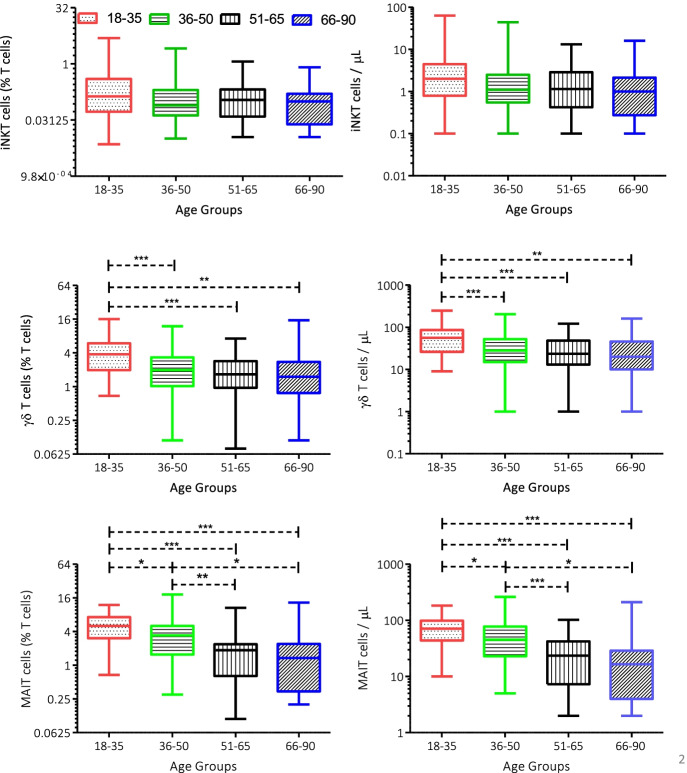
Percentages and absolute numbers of iNKT, γδ, and MAIT cells in peripheral blood of healthy adult individuals 18–35-year-old compared to 36–50-, 51–65-, and 66–90-year-old; 36–50-year-old compared to 51–65 and 66–90; 51–65 compared to 66–90 years old. *P* values (Kruskal–Wallis test-Dunn multiple comparison) are indicated inside the graphics (*p ≤ 0.05; **p ≤ 0.01; ***p ≤ 0.001)

The percentage and the absolute number of iNKT cells do not have any significant difference within the age groups, whereas γδ T cells and MAIT cells showed negative relation with the age (Fig. 2). iNKT cell percentage and absolute numbers showed no statistically significant difference between the different age groups (*p* = 0.0899 and *p* = 0.0516 respectively), whereas a slight dip (median) is observed between the age groups 18–35 (0.136) and 36–50 (0.078); however, later age groups recovered the differences (Fig. 2).

γδ T cell percentage and absolute numbers both showed a significant difference between different age groups *p* < 0.0001, whereas Dunn’s multiple comparisons revealed that the younger age group (18–35) has significantly higher γδ T cells in comparison to other age groups (36–50, 51–65, and 66–90); in addition, a significant difference was only achieved between the age group 18–35 vs 36–50, 51–65, and 66–90 (Fig. 2). Overall, median % γδ T cells decrease as increase in age groups, in age group 18–35, 3.78; in age group 36–50, 1.98; in age group 51–65, 1.66; and in age group 66–90, 1.5 respectively (Table 3).

MAIT cells also showed a statistically significant difference between the age groups for both percentages and absolute numbers *p* < 0.0001. Dunn’s multiple comparison showed not only the younger age groups (18–35) but middle-aged group (36–50) also have significantly higher MAIT cells in comparison to the older population (50–65 and 66–90) (Fig. 2). Median % MAIT cells decreases as the age groups increase, in age group 18–35, 5.02; in age group 36–50, 3.42; in age group 51–65, 1.85; and in age group 66–90, 1.34 respectively (Table 3).

### Correlation with age

**Fig. 3:**
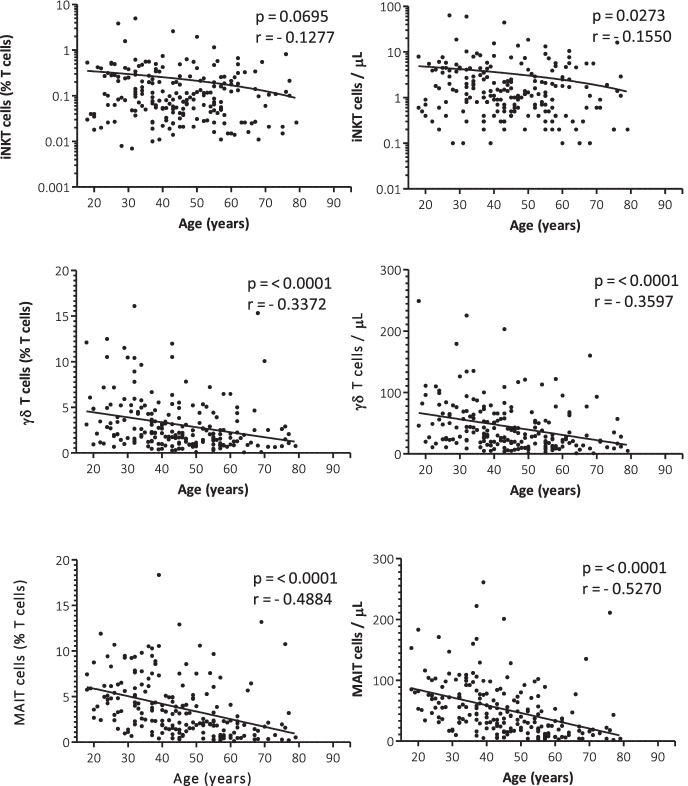
Correlation between age and the percentages and absolute number of iNKT cells, γδ T cells, and MAIT cells in adult healthy population. Spearman’s rank correlation coefficient and *p* values are indicated inside the graphics

iNKT cells (% T cell) did not show any correlation with age group (*p* = 0.0695, *r* =  − 0.1277); however absolute number of iNKT cells showed modest negative correlation (*p* = 0.0273, *r* =  − 0.1550). Both the percentage and the absolute number of γδ T cells and MAIT cells showed a stronger negative correlation with age (Fig. 3), concerning the relationship between various immunological parameters and ageing.

## Discussion

An enormous number of studies have been published in the last two decades highlighting the importance of unconventional T cells, and have been explored in different pathological conditions, such as infections (bacterial, viral, and fungal), immune-mediated diseases, autoimmune disorders, malignancies (solid and hematological), and cell-based immunotherapy. Characteristics of unconventional T cells are shown to be affected by age, gender, race and can lead to significant changes in the frequency and phenotype of these cells. However, there are very scanty studies done on healthy subjects to establish the reference range and it became very difficult to compare the results of patients because of the lacking of data and difference in previously published results.

There are very few studies performed on healthy individuals and to the best of our knowledge, our study is the only one that established the reference range for iNKT cells, γδ T cells, and MAIT cells together. The advantage of our study is that it is a single-center, prospective study covering iNKT cells, γδ T cells, and MAIT cells altogether, including homogeneous population (Caucasian), large number of gender-balanced samples, characterized with four age group–based distribution, and intensive exclusion criteria. Our results for iNKT cells were similar to those obtained by Montoya et al., from 90 healthy adult individuals: 40 males and 50 females [49], Peter et.al., from 10 healthy volunteers [50], Lucas et.al., from 11 normal healthy adults [51], and Sandberg et.al., in a cohort of 75 healthy individuals [52]. However, Fereidouni et al., in 40 healthy adults (24 males, 15 females; mean age 28 years) from Iran, reported a higher percentage of 6B11-iNKT cells among T lymphocytes compared to our results [53] (Tab s1). This higher percentage of iNKT cells can be explained by the fact that the mean age of their subjects was younger than the mean age of our subjects and the lower number of samples included in their study.

The majority of γδ T cells around 50–90% use Vγ9 and Vδ2 as a variable element in peripheral blood [54] and are most commonly referred to as γδ T cells. Our data are comparable and in agreement with previously published cohorts such as Michishita et al.’s study, on 120 healthy adult Japanese individuals (53 males and 64 females, mean age 40 years) [55] and Sonia Fonseca et al., on 30 Caucasian Portuguese healthy adults (18 males and 12 females, median age 47 years) [56] (Tab s2), whereas the same studies provide contradictory data while comparing to pan γδ T marker; Michishita et al., Sonia Fonseca et al., and Andreu-Ballester et al. reported significantly higher percentage and the absolute number of γδ T cells [55–57] (Tab s3).

Mucosa-associated invariant T cell frequency in PB is the highest among the unconventional T cells. Our results are in agreement with a similar cohort of 133 Korean healthy individuals age ranging from 21 to 92 years (48.1% females) by Lee et al. [58], and Novak et al., on 100 blood samples, age ranging from 0 to 100 years 50 male and 50 female [59] (Tab s4).

The frequency of cellular components of the immune system can change during ageing. In our study, we found that iNKT cells (median percentage and absolute numbers) do not follow immunosenescence and there is no statistically significant difference between the age groups. A notable slight decrease was observed in median values between the young (18–35) and middle age group (36–50); however, later the decrease was recovered in further age groups. Our data do not match with previously published cohorts by Jing et al. in 2007 on a total of 49 young (34 female and 15 male, median age 30.2 years) and 101 elderly individuals (76 female and 25 male, median age 78.1 years) using two sets of markers Vα24 and Vβ11 antibody pair and CD1d tetramer within lymphocytes because they found a statistically significant difference between young and elderly population [60]. Peralbo et al. obtained results on 25 young (mean of age was 29 years) and 25 elderly (mean of age was 77 years) individuals and found that Vα24 + Vβ11 + cells among lymphocytes were significantly lower in elderly subjects when compared to young individuals [61]. A similar study was published in 2001 by Delarosa et al., on 9 young (mean of age was 27 years) and 10 elderly (mean of age was 81 years); they found that the elderly population showed a decreased percentage of Vα24 + NKT cells compared to young population [62]. As expected, the percentage of iNKT cells did not show any correlation with age, whereas absolute iNKT cells have a minor correlation. Fereidouni et al. published a study on 40 samples, where the mean age of the group was 28 years and found a negative correlation between age and the percentage of iNKT cells.

There could be a few explanations to counter this discrepancy between our results and previously published results. All the aforementioned studies were done in previous decades and different clones of antibodies were used. 6B11 clone of iNKT cells specifically detects CDR3 region of the canonical Vα24Jα18 TCR rearrangement, and it was found to detect lower yet specific iNKT when 6B11 clone was used together with Vα24 within CD3 + T cells [49, 63]. In the study conducted by Jing et al., the representation of male versus female samples was unbalanced, which could distort the results. Our study was prospective with rigorous inclusion criteria and gender-balanced ratio, different age group samples are represented, and, last but not the least, sample size of the previous studies was lower compared to our study.

In contrast to iNKT cells, age-related changes in γδ T cells have been found in our study. Both percentages and absolute numbers showed a significant difference between the age groups. Percentages and the absolute number of γδ T cells are higher in younger age and show a slight but not significant decrease in middle-aged and older people. Similar results were published by Caccamo et al. in 2006 at 3 different age groups (2–15, 20–30, and 30–60 years) showing that γδ T cells gradually decrease beyond the age of 20–30 years and this depletion process with age appears to be rather slow and steady [64]. Several other studies also confirm that the number of γδ T cells decreases with age [55, 65]. Our results are similar to those previously published by Fonseca et al., who found that γδ T cell percentage and absolute number were negatively correlating with age. Few other studies also proved the negative correlation of γδ T cells with age [55, 56, 64, 65]. The advantage of our study is that it is a single-center, prospective study, including homogeneous population (Caucasian), large number of samples, characterized with four age group–based distribution, and intensive exclusion criteria.

MAIT cell frequency (percentages and absolute numbers) was found to be negatively correlated with the age group in our study. Lee et al. found that MAIT cell percentages were significantly lower in the middle and the elderly age group people than in the young group [58]. Chen et al. in 2019 published a study on a larger cohort including 379 healthy individuals (including 13 cord blood, 100 children (< 14 years old), 90 young-aged (20–40 years old), 88 middle-aged (41–60 years old), 88 elderly (above 60 years old)) and found that the percentage of MAIT cells progressively increased up to the young-aged group from cord blood; however, beyond young group, the frequency of MAIT cells decreases in older age groups [66]. Our results for MAIT cells were also in consensus with previously published studies that found a negative correlation of MAIT cells with age [59, 66].

Ageing is inevitable and various immune cells have been studied well in the context of immune ageing, leaving aside unconventional T cells. Like various immune cells, unconventional T cells also get adversely affected by age-related pathological conditions such as obesity, asthma, inflammatory bowel disease, diabetes, and cancers [67]. Cancer is an age-related disease; in the USA, 1.7% cancer-related deaths have been recorded in both genders who were < 40 years of age, whereas 90% of the cancers diagnosed in those are > 50 years of age [68]. The role of unconventional T cells in anti-tumor immunity was highlighted in various studies and a recent review by Nilberto et al. discussed the role of unconventional T cells in leukemia and anti-tumor immunity [69]. The age-related decrease in the number of unconventional T cells highlights the concern to age-related pathological conditions, however to establish direct association demands more detailed studies. In this study, we have established age-dependent reference ranges of unconventional T cells, which might be used as a foundational study for further research/clinical studies to compare unconventional T cells and age-related pathological conditions or disease progression and follow-ups.

In conclusion, this study is representative of the Caucasian population, considering the age-related reference ranges of unconventional T cells found in healthy adult individuals, which provide a window to explore various areas investigating immune-mediated pathological conditions and disease prognosis in clinical and research fields. In summary, γδ and MAIT cell frequency is higher in the younger age group than the elderly group and depicts a negative correlation with the age and supports immunosenescence. However, the frequency of iNKT cells has no statistical difference in the different age groups and a negative correlation with age was not observed.

## Supplementary Information

Below is the link to the electronic supplementary material.

Supplementary file1 (DOCX 118 KB)

## Data Availability

The data that supports the finding of the study is available from the corresponding author upon request.

## Abbreviations

iNKT cells: Invariant natural killer T cells
MAIT: Mucosa-associated invariant T cells
MHC: Major histocompatibility complex
TCR: T cell receptor
