# Supplementary material for: Age-dependent frequency of unconventional T cells in a healthy adult Caucasian population: a combinational study of invariant natural killer T cells, γδ T cells, and mucosa-associated invariant T cells
Source: GeroScience. 2022 Jan 17;44(4):2047–60. doi: 10.1007/s11357-022-00515-5 (PMC8763133; doi:10.1007/s11357-022-00515-5)
Supplement: Supplementary file 1 — Supplementary file1 (DOCX 118 KB) [file 11357_2022_515_MOESM1_ESM.docx]

Fig s1: Sample stability data for iNKT, γδ and MAIT cells performed on 10 healthy adults’ peripheral blood sample for 4 days consecutively.

Tab s1: Our results were compared with previously published studies, for iNKT cells

|  | iNKT cells | | | | |
| --- | --- | --- | --- | --- | --- |
| References | Sample size | % T cells (range) | % Mean ± SD | cells/µL (range) | Mean ± SD |
| Our results | 203 | 0.095 (0.007-4.94) | 0.2348 ± 0.5125 | 1.3 (0.1-63.5) | 3.334 ± 7.27 |
| Montoya et. al., 2006 | 90 | 0.01 - 0.92 | 0.17 ± 0.19 | NA | NA |
| Peter et.al., 2002 | 10 | 0.01 - 0.1 of PBMC | NA | NA | NA |
| Lucas, et.al, 2002 | 11 | 0.01 - 0.14 of PBMC | NA | NA | NA |
| Sandberg, et.al., 2003 | 75 | 0.05 (0.01 - 0.19) of PBMC | NA | NA | NA |
| Fereidouni et al., 2009 | 40 | 0.54 (0.14 - 1.78) | NA | NA | NA |

Tab s2: Our results were compared with previously published studies, for γδ T cells as a V δ2 marker

|  | γδ T cells | | | | |
| --- | --- | --- | --- | --- | --- |
| References | Sample size | % T cells (range) | % Mean ± SD | cells/µL (range) | Mean ± SD |
| Our results | 203 | 2.175 (0.078 - 16.9) | 3.119 ± 2.86 | 30 (1 to 249) | 44 ± 40.06 |
| Michishita et. al., 2011 | 120 | NA | NA | 1 - 153 | 43 ± 36 |
| Sonia Fonseca et. al., 2020 | 30 | NA | NA | 39 (6 - 243) | 63 ± 62 |

Tab s3: Our results were compared with previously published studies, for γδ T cells as a pan γδ T cell marker

|  | γδ T cells | | | | |
| --- | --- | --- | --- | --- | --- |
| References | Sample size | % T cells (range) | % Mean ± SD | cells/µL (range) | Mean ± SD |
| Our results | 203 | 2.175 (0.078 - 16.9) | 3.119 ± 2.86 | 30 (1 to 249) | 44 ± 40.06 |
| Michishita et. al., 2011 | 120 | NA | NA | 4 - 207 | 68 ± 44 |
| Sonia Fonseca et. al., 2020 | 30 | 4.3 (1.2 - 15.4) | 5.0 ± 3.6 | 63 (9 – 253) | NA |
| Andreu-Ballester et. al., 2012 | 157 | 3.13 (0.19 - 14.03) | NA | 69.8 (4.5 - 318.7) | NA |

Tab s4: Our results were compared with previously published studies, for MAIT cells

|  | MAIT cells | | | | |
| --- | --- | --- | --- | --- | --- |
| References | Sample size | % T cells (range) | % Mean ± SD | cells/µL (range) | Mean ± SD |
| Our results | 203 | 2.99 (0.11 - 18.36) | 3.801 ± 3.175 | 42 (2 – 261) | 52.88 ± 45.1 |
| Lee. et al, 2013 | 133 | 2.12 (0.19 - 21.7) | - | - | - |
| Novak et al, 2014 | 100 | 1.195 | - | 19 | - |
